# Moving through Life-Space Areas and Objectively Measured Physical Activity of Older People

**DOI:** 10.1371/journal.pone.0135308

**Published:** 2015-08-07

**Authors:** Erja Portegijs, Li-Tang Tsai, Taina Rantanen, Merja Rantakokko

**Affiliations:** Gerontology Research Center and Department of Health Sciences, University of Jyvaskyla, Jyväskylä, Finland; University of Edinburgh, UNITED KINGDOM

## Abstract

**Objectives:**

Physical activity–an important determinant of health and function in old age–may vary according to the life-space area reached. Our aim was to study how moving through greater life-space areas is associated with greater physical activity of community-dwelling older people. The association between objectively measured physical activity and life-space area reached on different days by the same individual was studied using one-week longitudinal data, to provide insight in causal relationships.

**Methods:**

One-week surveillance of objectively assessed physical activity of community-dwelling 70–90-year-old people in central Finland from the “Life-space mobility in old age” cohort substudy (N = 174). In spring 2012, participants wore an accelerometer for 7 days and completed a daily diary including the largest life-space area reached (inside home, outside home, neighborhood, town, and beyond town). The daily step count, and the time in moderate (incl. walking) and low activity and sedentary behavior were assessed. Differences in physical activity between days on which different life-space areas were reached were tested using Generalized Estimation Equation models (within-group comparison).

**Results:**

Participants’ mean age was 80.4±4.2 years and 63.5% were female. Participants had higher average step counts (p < .001) and greater moderate and low activity time (p < .001) on days when greater life-space areas were reached, from the home to the town area. Only low activity time continued to increase when moving beyond the town.

**Conclusion:**

Community-dwelling older people were more physically active on days when they moved through greater life-space areas. While it is unknown whether physical activity was a motivator to leave the home, intervention studies are needed to determine whether facilitation of daily outdoor mobility, regardless of the purpose, may be beneficial in terms of promoting physical activity.

## Introduction

Outdoor physical activity, particularly walking, plays a key role in maintaining health and mobility in old age [1–3]. Physical activity refers to activities performed in daily life and includes activities such as those related to occupation, leisure-time, housework and transportation. The majority of moderate intensity physical activity in older adults occurs outside of the individual’s home [4,5]. Furthermore, a higher frequency of leaving the home is beneficial for maintaining health and function [6–8], and being able to go beyond the own neighborhood has been associated with positive health outcomes, such as higher physical and cognitive function [6–9].

Moving through a greater life-space area, that is, a spatial area in which a person commonly acts, enables an individual to participate in meaningful activities in the society [9–11]. These activities may include physical activities or exercise, which may explain at least part of the association found between higher physical activity and higher life-space mobility [9,12]. Life-space mobility reflects the spatial area an individual moves through, the frequency of movement and their need for assistance [13]. Optimal mobility of an individual may involve motorized transport in addition to walking [14], which is a physical activity. When moving through greater life-space areas, an individual is more likely to use a car or other forms of motorized transportation [9,15,16]. Therefore, it is not known whether moving through greater life-space areas, such as the town or beyond, is associated with higher physical activity.

Individuals going outdoors less frequently or moving through smaller life-space areas likely have poorer health and function, and coexisting lower levels of physical activity [6–8,17]. It is not known whether the association between life-space mobility and physical activity [9] is merely explained by accumulation of poor outcomes within an individual (between-subject variation) or whether moving through greater life-space areas increases an individual’s physical activity on that day (within-subject variation). The aim of the current study was to determine whether going outdoors and moving through greater life-space areas is associated with greater physical activity of community-dwelling older individuals. Instead of exploring associations by comparing groups with different characteristics, one-week longitudinal data (within-subject analyses) were used to study the relationship between objectively assessed physical activity and life-space areas reached on different days by the same individual, in order to provide further insight in potential causal relationships.

## Materials and Methods

### Study design and recruitment

One-week surveillance of objectively assessed physical activity as part of the substudy of the “Life-space mobility in old age” (LISPE) cohort study in community-dwelling, 75–90-years-old people, living in the municipalities of Muurame and Jyväskylä in central Finland. Study methods were published previously [18]. Baseline data were collected during an interview in the participants’ homes (N = 848). Eligibility for participation (living independently, able to communicate, residing in the recruitment area and willing to participate) was determined during an initial phone interview. From March 26th to June 15th, 2012, a tri-axial accelerometer was offered to a subgroup of participants, of whom 190 agreed to participate [18]. Participants signed a written informed consent form. LISPE was approved by the Ethical Committee of the University of Jyväskylä, Finland, in October 2011.

### Accelerometer and diary

An accelerometer (Hookie, tri-axial, “AM20 Activity Meter”, Hookie Technologies Ltd, Espoo, Finland) was provided with detailed written and oral instructions. The accelerometer was worn on the right hip for 7 consecutive days following the face-to-face interview and returned by prepaid mail or if necessary picked up. Participants were instructed to wear the accelerometer daily from waking up to going to sleep, removing it for water activities only.

Accelerometer default settings for thresholds and formulas for calculating different parameters supplied by the manufacturer were used (Hookie Technologies Ltd, Espoo, Finland). The sampling frequency was 100 Hz and the measurement range of the device is ± 15 g_0_ (gravity of the earth). Activities were identified based on rhythmic accelerations and intensity [19]. Walking (rhythmic moderate intensity (±2g)), running (rhythmic higher intensity (>±4g)) and other activities (without rhythm moderate to higher intensity (>±2.5g)) were identified and merged into one category “*moderate activity*”, due to little running and other activity time. Additionally, *low activity* (without rhythm low intensity (<±2.5g)) and *sedentary behavior* (no activity detected for ≥5s) were identified. Total time in moderate and low activity or sedentary behavior, and total *step count* of each day were analyzed.

The total daily *wear time* of the accelerometer was calculated from the self-report diary data. Participants were asked to keep a diary in which they registered the time when the accelerometer was put on and taken off as well as potential breaks in which the accelerometer was taken off. To retain as many participants in the analyses as possible, missing accelerometer wear time values were imputed with the average wear time of that respective individual (if missing 1 or 2 days; N = 15) or the group average of each day (if missing for all days; N = 1). Sensitivity analyses did not reveal a marked effect of the imputation. In addition imputation is justified, as previous literature shows that wear time mainly affects sedentary time and not activity time [20, 21]. Participants also recorded daily in the diary the greatest *life-space area* they moved through (1) home, 2) outside home, 3) neighborhood, 4) town, 5) beyond; in accordance with the Life-Space Assessment [13]). Unfortunately, no information regarding trips, duration in each life-space area or mode of transportation used was available. The *number of days moving beyond the neighborhood* (that is, reaching the town or beyond town area) during the assessment week was calculated and categorized in tertiles; 0–3, 4–5, and 6–7 days.

To ensure that the variation in physical activity in older populations was captured [22], participants were excluded if there were less than 4 valid accelerometer days (N = 11) or >1 days in-between consecutive measurement days (N = 1) [18]. A valid day was defined as a day with ≥10 hours of accelerometer wear time. Few days were omitted due to technical errors of the accelerometer (N = 3) or loss of the accelerometer in the mail (N = 1) [18]. In total, data of 174 participants were analyzed.

### Other measures

The demographic variables *age* and *gender* were derived from the national register. Type of *neighborhood* (urban vs. rural) and *housing* (apartment block, row house, and semi-detached or detached house) were determined based on observation by the interviewers. Other variables were obtained using self-report questionnaires. The frequency of use of *transportation* modes (car driving, car passenger, public transportation, or taxi or Special Transportation Service) was assessed and dichotomized into at least few times a month vs. less frequently / never. The number of self-reported *chronic diseases* was calculated from a list of 22 physician diagnosed chronic diseases and an additional open-ended question about any other physician diagnosed chronic conditions [23]. Perceived *difficulty in walking 2 km* (no vs. some / a great deal of difficulty / unable) was assessed, reflecting an individual’s abilities in relation to the environment he or she moves through.

### Statistical analysis

Day totals of each assessment day and overall day averages were computed for physical activity measures: (sum / number of valid days). Sedentary time was corrected for accelerometer non-wear time; (total wear time–(moderate + low activity time)). In addition, time proportions in each physical activity were computed: (time in activity / total wear time)*100%. Within-subject variation in physical activity was calculated as relative ratio ((minimal / maximal)*100%) and within-subject variation in life-space area as absolute difference (greatest–smallest life-space area reached). Spearman correlation coefficients of the within-subject variation in physical activity and life-space area were computed.

Differences in participant characteristics in the groups according to the number of days that a participant moved beyond the neighborhood during one week were tested with Kruskal Wallis- and Chi-square-tests. Factors with a statistically significant difference between the groups were considered potential confounders in subsequent analyses.

Group differences in physical activity measures *between participants* moving beyond the neighborhood on 0–3, 4–5, or 6–7 days were tested with Generalized Linear Models (between-subject variation). Physical activity measures were transformed using log link transformation, due to skewed distribution. Analyses estimating step counts, moderate and low activity time, and sedentary time were adjusted for accelerometer wear time. Analyses on time proportions in moderate and low activity or sedentary behavior were unadjusted. All analyses were repeated and further adjusted for age and gender, and potential confounders.

Statistical significance of the *day-to-day variation* (within-subject) in physical activity measures due to differences in life-space area reached was tested; within each individual, days on which the same life-space area was reached were clustered. The variation in physical activity (dependent variable with log link transformation) according to the life-space area (between-subject factor) reached on that respective day was tested using General Estimating Equation (GEE) models with unstructured working correlation matrix. Day of assessment was included as within-subject factor. All participants moving through at least two different life-space areas during the assessment week (N = 150) were included in the analyses. GEE modeling was conducted with and without multivariate imputation by chained equations (MICE) procedure [24] in SPSS (version 20.0; IBM, Armonk, NY, USA). The results of these analyses were similar; only the imputed data are reported. Analyses estimating step counts, moderate and low activity time, and sedentary time were adjusted for accelerometer wear time. Subsequently, the analyses were further adjusted for age, sex, and potential confounders. The life-space area “neighborhood” was used as reference group in the analyses as this area most likely encompasses activities that contribute to the accumulation of physical activity. Of the 150 participants included in these analyses, 59% visited this area at least once during the assessment week. Supplementary analyses were conducted using the life-space area “inside only” as the reference group; 20% of the included participants restricted their life-space area at least once to the home. Finally, for each individual, the physical activity scores were determined for the life-space areas reached (means in case of recurrence) and used to calculate the median score and interquartile range of the physical activity in the different life-space areas. Statistical significance was set at P < .05.

## Results

### Between-subject analyses

Participants were on average 80.4 ± 4.2 years-old and 63.5% was female. Table 1 shows that a higher weekly frequency of moving beyond the neighborhood was associated with younger age. Men, participants without walking difficulty, those driving a car at least monthly, or those using taxi or Special Transportation Services were overrepresented in the groups with a higher weekly frequency of moving beyond the neighborhood. Accordingly, subsequent analyses were adjusted for age, sex, walking difficulty, and monthly car driving. Use of taxi or Special Transportation Services was not adjusted due to relatively low numbers of participants.

**Table 1:** Participant Characteristics according to the Number of Days Moving beyond the Neighborhood during One Week.

	0–3 days (N = 53)		4–5 days (N = 49)		6–7 days (N = 72)		
	Median	IQR	Median	IQR	Median	IQR	P^a^
**Age** (yrs)	81.8	(7.1)	79.6	(7.2)	78.0	(5.5)	**.001**
**Chronic diseases** (n)	4.0	(3)	4	(3.5)	4	(4)	.636
**Accelerometer wear time** (hr)	13.6	(2.0)	13.7	(2.5)	14.3	(1.9)	**.035**
	**%**	**n**	**%**	**n**	**%**	**n**	**P** ^b^
**Gender** (female)	81	(43)	65	(32)	50	(36)	**.002**
**Walk difficulty 2km** (yes)	57	(30)	27	(13)	15	(11)	**< .001**
**Car driver** (yes)	19	(10)	53	(26)	82	(40)	**< .001**
**Car passenger** (yes)	70	(37)	61	(30)	60	(43)	.484
**Public transport** (yes)	38	(20)	39	(19)	39	(28)	.990
**Taxi** (yes)	66	(18)	10	(5)	11	(8)	**.001**
**Neighborhood** (Urban)	94	(50)	92	(45)	96	(69)	.650
**Housing**							.693
(Apartment block)	55	(29)	49	(24)	46	(33)	
(Row house)	13	(7)	20	(10)	24	(17)	
Semi-detached / detached house)	32	(17)	31	(15)	31	(22)	

IQR = interquartile range.

^a^ Kruskal Wallis.

^b^ Chi-square tests.

Table 2 shows that participants moving beyond the neighborhood more frequently reached higher step counts, greater moderate activity and lesser sedentary time. After adjustment for accelerometer wear time, age, sex, walking difficulty, and monthly car driving the group differences were somewhat attenuated, and for sedentary behavior the associations did not remain statistically significant.

**Table 2:** Group Comparisons of Physical Activity according to the Number of Days Moving beyond the Neighborhood.

	0–3 days (N = 53)		4–5 days (N = 49)		6–7 days (N = 72)				
	M	95%CI	M	95%CI	M	95%CI	P^a^	P^b^	P^c^
**Step counts** (1000*n)	1.7	1.3–2.2	2.1	1.5–2.7	4.1	3.2–5.2	**< .001**	**< .001**	**.001**
**Moderate activity time** (min)	16.7	13.2–21.2	19.6	15.3–25.0	36.6	29.8–44.8	**< .001**	**< .001**	**.001**
**Moderate activity** (%) ^d^	2.0	1.6–2.6	2.4	1.9–3.0	4.4	3.6–5.4	**< .001**	**< .001**	**.002**
**Low activity time** (hr)	2.4	2.2–2.7	2.6	2.4–2.9	2.8	2.6–3.0	.060	.068	.490
**Low activity** (%) ^d^	18.0	16.4–19.7	19.1	17.4–21.0	20.3	18.7–21.9	.205	.201	.734
**Sedentary time** (hr)	10.8	10.5–11.1	10.6	10.3–10.9	10.1	9.9–10.4	**.003**	**.004**	.085
**Sedentary** (%) ^d^	79.4	77.2–81.7	77.1	74.8–79.4	72.7	70.9–74.5	**.008**	**.018**	.181

M = Mean, 95%CI = 95% confidence interval.

^a^ Generalized Linear Models adjusted for accelerometer wear time.

^b^ Generalized Linear Models adjusted for accelerometer wear time + age + sex.

^c^ Generalized Linear Models adjusted for accelerometer wear time + age + sex + walking difficulty + monthly car driving.

^d^ Not adjusted for accelerometer wear time in any model.

**Fig 1:**
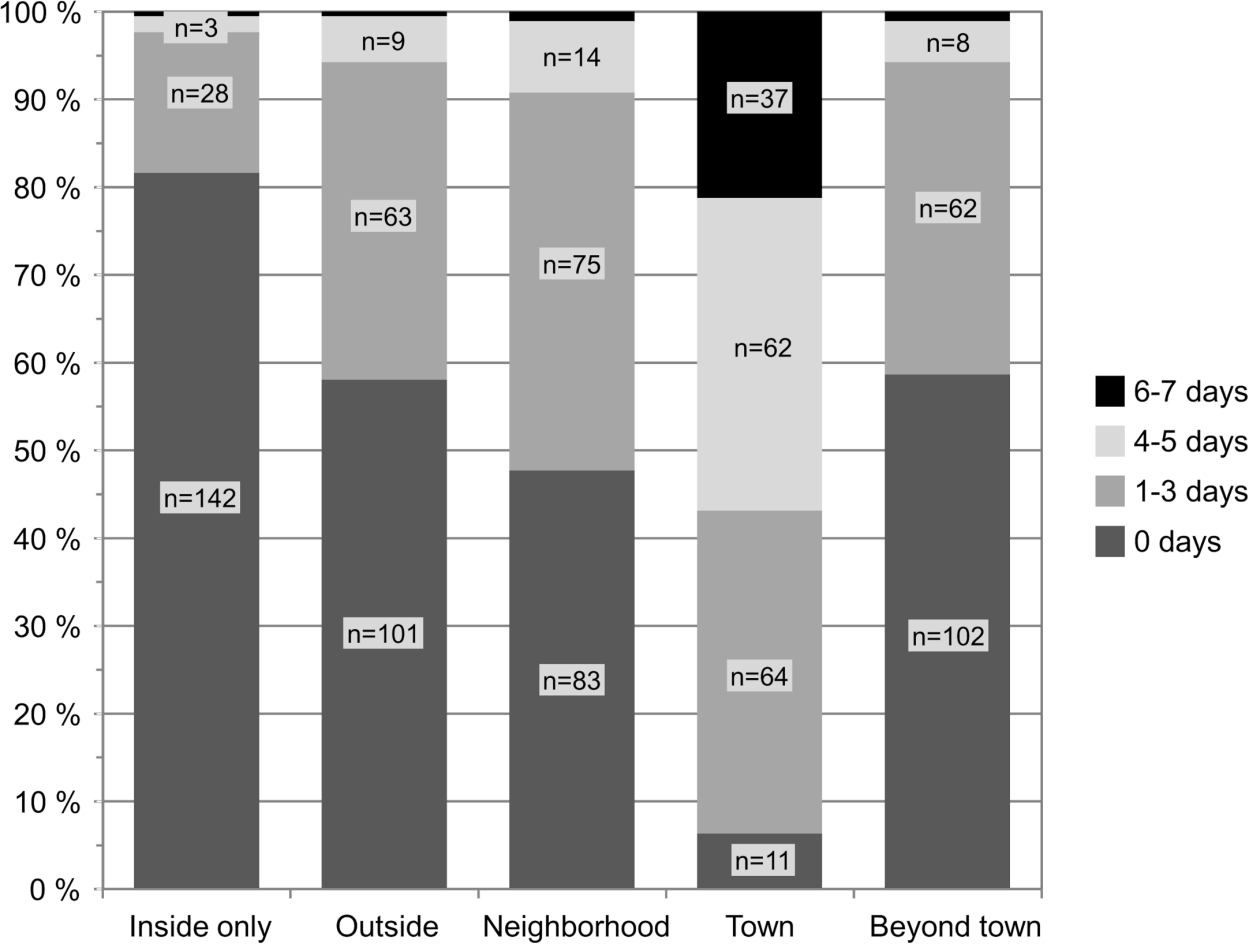
Proportion of Participants according to Greatest Life-Space Area and Frequency of Reaching Respective Area (N = 174). NOTE: Moving in the life-space areas inside (n = 1), outside (n = 1), neighborhood (n = 2) or beyond town (n = 2) for 6–7 days a week.

Fig 1 shows that nearly all participants (94%) reached the town area at least once in the assessment week, but the majority of them on multiple days of the week. Eighteen percent of the participants stayed within the home on at least one day.

All physical activity measures showed large within-subject variation. Larger within-subject variation in life-space area was associated with larger variation in step counts (Rs = -.35, p < .001) and moderate activity time (Rs = -.28, p < .001). There was no association between within-subject variation in life-space area and the variation in low activity time (Rs = -.11, p = .139) or sedentary time (Rs = .11, p = .130).

### Within-subject analyses

**Fig 2:**
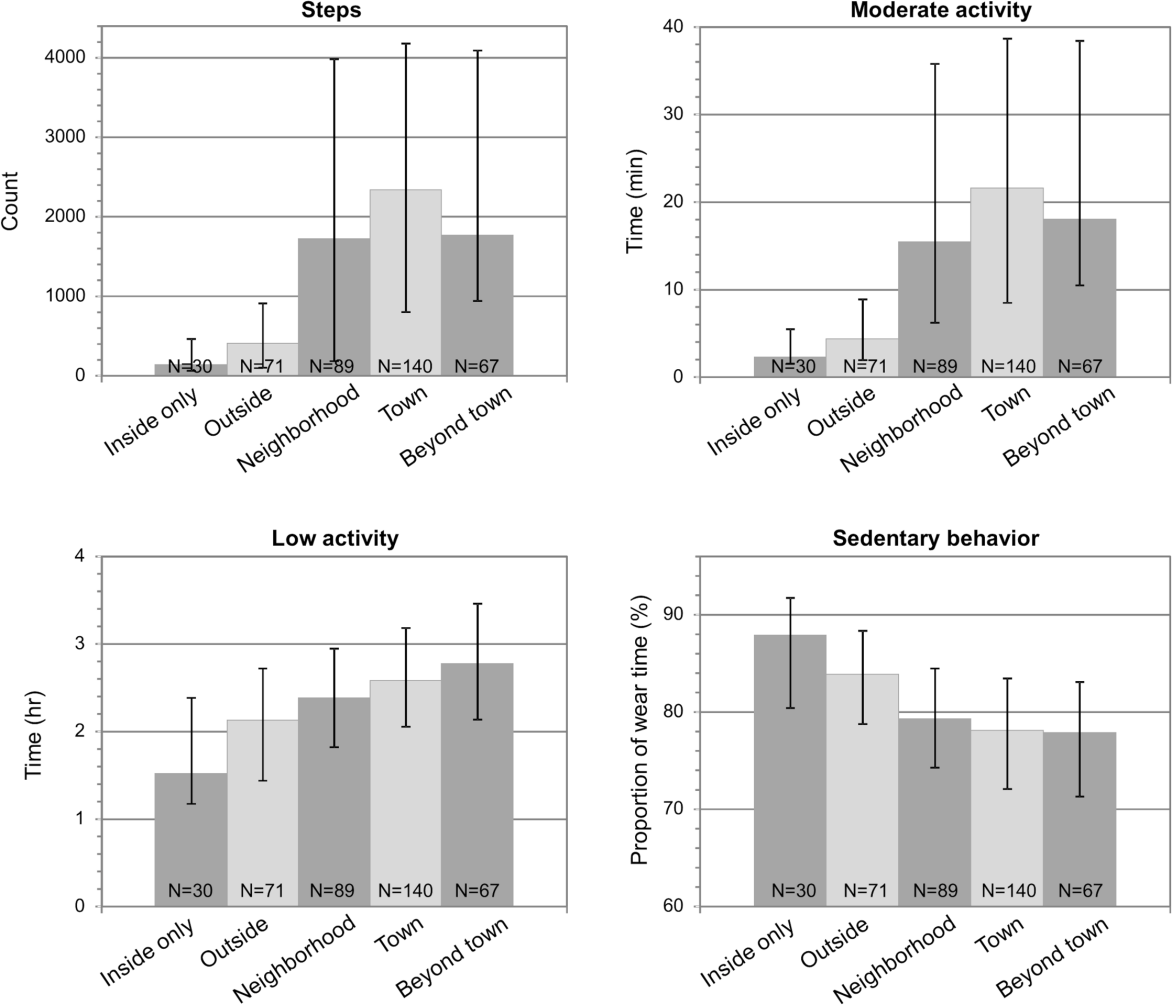
Median (and Interquartile Range) Physical Activity according to the Life-Space Area Reached by the Participant on the Same Day (N = 150). NOTE: Includes only data from participants who reached at least two different life-space areas during the assessment week, the number of people reaching each area is indicated.

Fig 2 showed that on days when a participant moved through a greater life-space area more physical activity was detected. Table 3 shows the results of the GEE models. Compared to a day when a participant stayed within their own neighborhood, staying inside the home was associated with lower physical activity and going to the town area was associated with greater physical activity in all measures. However, when moving beyond the town, step count and moderate activity time were not different from moving in the neighborhood only. Yet, low activity time was statistically significantly greater and sedentary time significantly lower when moving beyond the town. When the GEE models were further adjusted for age, sex, walking difficulty and car driving, differences in physical activity associated with the life-space areas were attenuated but the overall trend remained statistically significant (p < .001). Compared to a day when a participant stayed inside the home, going outside was associated with significantly greater physical activity (p≤.033), except for low activity time (p = .091).

**Table 3:** Within-Subject Comparison of Days on which a Participant Reached Different Life-Space Areas (N = 150).

	Inside only		Outside		Neighborhood	Town		Beyond town		
	b	95%CI	b	95%CI	b	b	95%CI	b	95%CI	P
**Step counts** (1000*n) ^a^	**-.63**	**-.80 –- .45**	**-.31**	**-.42 –- .19**	0	**.20**	**.10 – .28**	.06	-.11 – .23	**< .001**
**Moderate activity time** (min) ^a^	**-1.23**	**-1.55 –- .91**	**-.47**	**-.66 –- .28**	0	**.29**	**.16 – .41**	.10	-.12 – .32	**< .001**
**Moderate activity** (%)	**-1.24**	**-1.57 –- .91**	**-.46**	**-.65 –- .27**	0	**.28**	**.15 – .41**	.09	-.12 – .31	**< .001**
**Low activity time** (hr) ^a^	**-.16**	**-.27 –- .04**	-.05	-.11 – .01	0	**.05**	**.01 – .10**	**.12**	**.05 – .19**	**< .001**
**Low activity** (%)	**-.16**	**-.27 –- .05**	-.04	-.10 – .03	0	**.05**	**.00 – .10**	**.10**	**.04 – .17**	**< .001**
**Sedentary time** (hr) ^a^	**.05**	**.02 – .07**	**.02**	**.003 – .03**	0	**-.02**	**-.03 –- .01**	**-.03**	**-.05 –- .01**	**< .001**
**Sedentary** (%)	**.05**	**.02 – .07**	**.02**	**.00 – .03**	0	**-.02**	**-.03 –- .01**	**-.02**	**-.04 –- .00**	**< .001**

NOTE: General Estimating Equation models including participants who reached at least two different life-space areas during the assessment week. Statistically significant differences (p < .05) from average day “neighborhood” are indicated in bold.

b = regression coefficient, 95%CI = 95% confidence interval.

^a^ Adjusted for accelerometer wear time.

## Discussion

This study expands the current knowledge on the association between life-space mobility and physical activity [9] and the importance of leaving the home frequently in old age [1,6–8]. To our knowledge this is the first study utilizing a longitudinal design that provides new insights in potential causal relationships. This study demonstrated that community-dwelling older people were more physically active on days when they go outdoors, and moving through greater life-space areas further increased their physical activity. Moving beyond the own town, however, did not increase step count or moderate activity time, probably because passive modes of transportation are used [9,16,25], but low activity time still increased. Conversely, the amount of sedentary time progressively decreased with each greater life-space area. From a public health perspective it is important to note that greater amount of time engaged in physical activity [3,26,27], even at low intensity, and lesser sedentary time [26,27] provide important benefits for maintaining health and function in old age.

Previous studies have shown large inter-individual and intra-individual variation of physical activity in older populations [22,28]. The current findings demonstrate that total variation in physical activity was at least partly related to moving through different life-space areas. In addition, individuals moving more frequently in the town area or beyond were more physically active. More frequent trips in greater life-space areas may indicate better general health and functioning [6–8], yet, adjusting the analyses did not mitigate the associations found. Combined with the results of the General Estimating Equation models, our results suggest that leaving the home and moving through greater life-space areas may increase physical activity. While other studies have shown that activities such as shopping are associated with higher levels of physical activity [29], physical exercise may also be the purpose for an individual to leave the home [5,29]. Consequently, intervention studies are needed to confirm the causal relationship and to determine whether facilitation of outdoor mobility may be beneficial in terms of promoting physical activity, regardless of the purpose when leaving the home.

Activities attended by an individual may differ according to the life-space area. For example, when moving around the home, outside or in the neighborhood, an individual is likely to use walking or other active forms of transportation to move oneself [9,15,16], thus activities that likely include moderate physical activity. This is in accordance with our findings that the amount of physical activity increased with each greater life-space area reached, from the home up to the town area. Walking, for transportation and recreational purposes, accounts for a major part of the daily physical activity in older individuals [2,3,26]. Only low activity, that accumulates in activities of daily living and comprises the main portion of physical activity in older people [3], continued to increase also when moving beyond the town area [9,16,25]. This seems in line with previous studies showing that being able to drive and using public transportation was associated with higher physical activity in older adults [5,30].

Intervention strategies targeting an increase in physical activity may improve health and functioning [27,31], but environmental design may also provide opportunities to increase physical activity in older individuals [32,33]. A growing number studies shows that attractive (e.g. greens spaces and esthetics) and walking friendly environments (e.g. including sidewalks and safe road crossings) are associated with higher physical activity in older people [34,35], and more frequent trips outside of the home [36–38]. Thus increasing outdoor mobility may provide a means to increase physical activity in older people. Yet, also the purpose and destination of the trip contribute to the mode of transportation chosen [15,16] and total amount of physical activity [5,29]. Unfortunately, we did not have data on where exactly the physical activity took place, the number of trips our participants made during any day, the travel mode used or the time spent in each life-space area. Thus it remains unknown how and in which life-space area the physical activity actually accumulated.

Strengths of this study include the use of objective measures of physical activity [28], that were available for at least 4 days, but in the majority of participants for 7 days. At least 4 days is necessary to capture the variation in physical activity in older populations, while more days are preferable [22]. The use of a tri-axial accelerometer allowed for comparisons of step counts and intensity-specific physical activity measures [28]. Although the study participants represent rather well-functioning older people, which is a commonly recognized limitation in aging research [39], there is large heterogeneity in the sample regarding age and sex as well as mobility function [18].

While a growing amount of studies have used accelerometers to measure physical activity also in older people, some concerns remain, especially regarding the sensitivity of accelerometers in detecting steps and activity in those walking at lower walking speed [28,40,41]. This likely leads to a slight underestimation of the total physical activity, but it is reasonable to assume that activity detected by accelerometers is likely to be beneficial for health [40]. The fact that we did not take into account duration of individual bouts of physical activity on the other hand may lead to a slight overestimation of the moderate activity time. This limitation may not be as obvious in older people, who generally display a pattern of intermittent and unstructured physical activity [28]. Accelerometer wear time in our study was self-reported. While a recall bias may be troublesome in studies of older people, we as well as other researchers [21] have confidence that the values are accurate. Determining wear time based on accelerometer data is also prone to bias, especially in older people who have longer periods of inactivity [20,21]. Furthermore, accelerometer wear time estimates affect mainly the time in sedentary behavior and less the time in activity [20,21]. Finally, most participants reached two to three life-space areas and consequently the within-subject analyses results need to be interpreted with caution.

## Conclusions

Community-dwelling older people are more physically active on days that they go outdoors, and moving through the neighborhood and town further increases their level of physical activity. However, once an individual moves beyond their town, only low activity time continues to increase, which may still have important health benefits. While it is unknown whether physical activity was a motivator for participants to leave the home, intervention studies are needed to determine whether facilitation of the frequency of leaving the home and moving through greater life-space areas may be beneficial in terms of promoting physical activity in older people. More detailed analyses, e.g. on individual trips made and travel modes, may provide insight into mechanisms underlying the accumulation of physical activity in older people in relation to the environment and life-space.
